# Supplementary material for: Agroindustrial Wastes as a Support for the Immobilization of Lipase from Thermomyces lanuginosus: Synthesis of Hexyl Laurate
Source: Biomolecules. 2021 Mar 17;11(3):445. doi: 10.3390/biom11030445 (PMC8002546; doi:10.3390/biom11030445)
Supplement: Supplementary file 1 [file biomolecules-11-00445-s001.pdf]

Agroindustrial Wastes as a Support for the Immobilization of Lipase from *Thermomyces lanuginosus*: Synthesis of Hexyl Laurate

Regiane K. de S. Lira [1], Rochele T. Zardini [1], Marcela C. C. de Carvalho [1], Robert Wojcieszak [2], Selma G. F. Leite [1] and Ivaldo Itabaiana Jr [1,2,*]

[1] Federal University of Rio de Janeiro, Department of Biochemical Engineering, School of Chemistry, RJ, 21941-909, Brazil; regianekessias@eq.ufrj.br; rochelezar@gmail.com; marcela.caetano18@gmail.com; selma@eq.ufrj.br
[2] Univ. Lille, CNRS, Centrale Lille, Univ. Artois, UMR 8181 – UCCS – Unité de Catalyse et Chimie du Solide, F-59000 Lille, France; robert.wojcieszak@univ-lille.fr
* Correspondence: ivaldo@eq.ufrj.br; Tel.: +552139387580

Suplementary Material

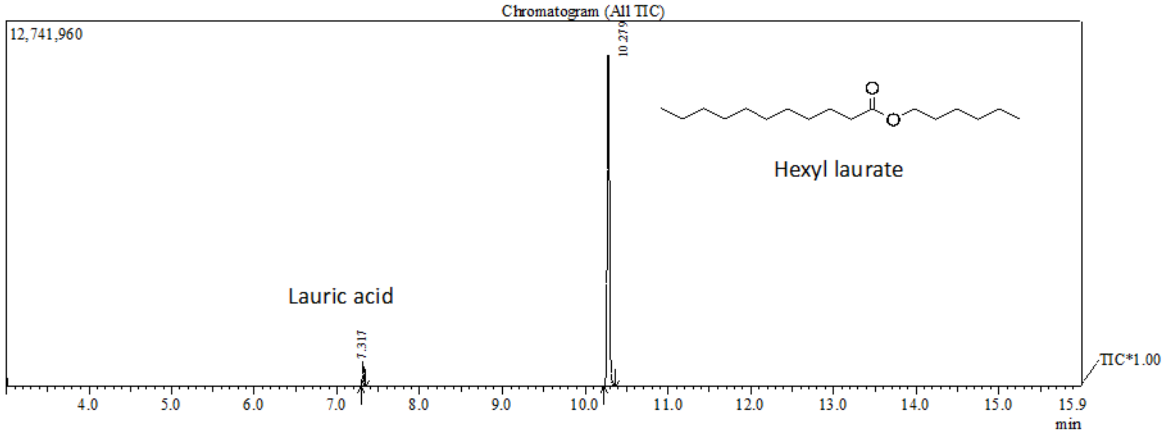

Peak#	Ret.Time	Start Tm	End Tm	m/z	Area	Area%	Height	Height%
1	7.317	7.292	7.342	TIC	986445	4.27	906371	7.26
2	10.279	10.225	10.367	TIC	22138313	95.73	11583600	92.74

Figure SI – GC-MS analysis of hexyl laurate catalyzed by BT-TLL biocatalyst at 240 min.

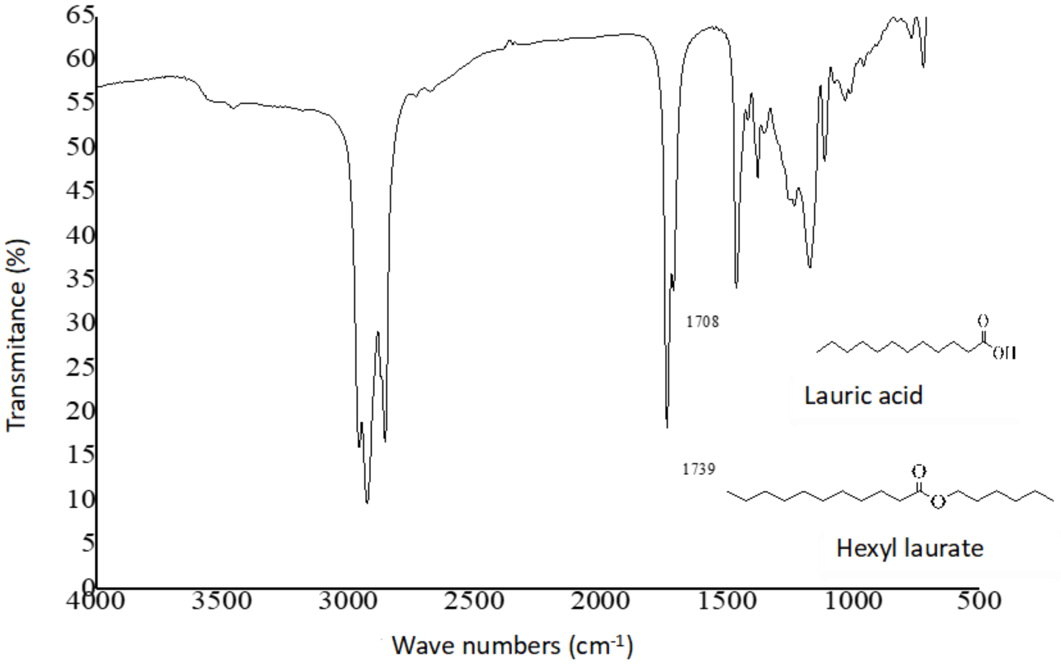

Figure S2 – FTIR spectrum of hexyl laurate catalyst by BM-TLL.

Table S1 – Chemical composition of agro-industrial wastes studied. CG- coffee grounds; SC - sugarcane bagasse; BM - babassu mesocarp, CC - corn cobs and RH - rice husks

Chemical composition (%)										
**Support**	**CNHS (%)**				**Cellulose**	**Hemicellulose**	**Lignin**	**Free Protein**	**Umidity (%)**	**Ash (%)**
	**C**	**N**	**H**	**S**						
CG	57,36 ±0,4	3,87 ±0,41	6,64 ±0,14	<1	7,69 ±0,6	30,78 ±0,1	28,11 ±0,5	0,25 ±0,1	6,68 ±0,1	0,38 ±0,0
SC	44,43 ±0,1	1,40 ±0,51	5,61 ±0,24	<1	32,38 ±0,1	12,99 ±0,0	21,34 ±0,5	0,07 ±0,1	4,66 ±0,1	14,79 ±0,1
BM	40,23 ±0,6	2,39 ±0,18	5,52 ±0,64	<1	50,25 ±0,3	8,77 ±0,7	7,90 ±0,3	0,12 ±0,1	9,62 ±0,1	0,35 ±0,3
CC	43,66 ±0,7	2,84 ±0,46	5,58 ±0,52	<1	33,38 ±0,3	31,72 ±0,6	8,55 ±0,1	0,09 ±0,1	9,56 ±0,1	0,57 ±0,1
RH	42,35 ±0,2	2,56 ±0,05	4,91 ±0,52	<1	37,10 ±0,9	12,53 ±0,6	18,08 ±0,1	0,13 ±0,1	9,94 ±0,1	0,52 ±0,1
